# Comprehensive Transcriptomic Analyses of Silk-Associated Genes and Functional Characterization of Key Silk Fibroins in *Plutella xylostella*

**DOI:** 10.3390/ijms26072842

**Published:** 2025-03-21

**Authors:** Rui-Chang Niu, Fan-Xin Meng, Qing-Hui Zeng, Yi-Jing Wang, Tong-Xian Liu, Dong Chu, Shi-Ze Zhang

**Affiliations:** 1 State Key Laboratory of Crop Stress Biology for Arid Areas, Key Laboratory of Plant Protection Resources and Pest Management of Ministry of Education, Key Laboratory of Integrated Pest Management on Crops in Northwestern Loess Plateau of Ministry of Agriculture and Rural Affairs, College of Plant Protection, Northwest A&F University, Yangling 712100, China; niurcyx@163.com (R.-C.N.); mengfanxin@nwafu.edu.cn (F.-X.M.); 18311516405@163.com (Q.-H.Z.); 19591283320@163.com (Y.-J.W.); 2 Institute of Entomology, College of Agriculture, Guizhou University, Guiyang 550025, China; tx.liu@gzu.edu.cn; 3 College of Plant Health and Medicine, Qingdao Agricultural University, Qingdao 266109, China; chinachudong@sina.com

**Keywords:** *Plutella xylostella*, transcriptomics, silk fibroin genes, amino acid composition, expression profiles

## Abstract

The diamondback moth (DBM), *Plutella xylostella* (Lepidoptera: Plutellidae), is a serious agricultural pest that utilizes silk as a defensive mechanism, with silk fibroins playing a pivotal role in this process. Through comprehensive transcriptomic analyses, we identified 3452 differentially expressed genes (DEGs) co-expressed in the silk gland of *P. xylostella* and associated with silk production. The Gene Ontology (GO) analysis revealed enrichment in categories related to protein synthesis, secretion, and extracellular matrix organization, while Kyoto Encyclopedia of Genes and Genomes (KEGG) analysis linked these genes to amino acid metabolism and protein processing pathways. Additionally, we identified three key silk fibroin genes: silk fibroin heavy chain (*FibH*), silk fibroin light chain (*FibL*), and fibrohexamerin (*P25*). We characterized the structure of these genes and analyzed the phylogenetic relationships, amino acid composition, hydrophilicity, and other physicochemical properties of the encoded silk fibroin proteins. The expression profiles revealed peak expression levels of these genes in the silk glands of fourth instar larvae. This integrative study enhances our understanding of the molecular mechanisms underlying silk production in *P. xylostella* and provides a foundation for future research into the biological roles, evolutionary trajectories, and potential applications of these silk fibroin genes in agricultural pest management and biotechnology.

## 1. Introduction

The diamondback moth (DBM), *Plutella xylostella* (Lepidoptera: Plutellidae), is a major pest of cruciferous vegetables worldwide, causing significant damage to crops [1]. The larvae of DBM damage leaf tissues, resulting in notches and holes [2]. Due to its high fecundity, short life cycle, and strong adaptability, this pest is responsible for billions of dollars in annual economic losses [3]. However, traditional chemical control methods are becoming less effective as DBM develops resistance to various insecticides, including chlorantraniliprole, beta-cypermethrin, and indoxacarb [4,5]. As a result, there is an increasing need for the development of innovative pest control strategies, which is currently a major research focus.

The silk gland plays a crucial role in silk-producing insects, as it is responsible for producing and releasing silk used for various purposes, such as nest construction, predator evasion, temperature regulation, and attachment [6,7,8]. For instance, silkworm larvae spin silk to create protective cocoons that shield them from natural enemies and UV radiation [9]. In addition to cocoon formation during pupation, DBM exhibits a unique defense behavior by spinning silk to escape threats [10,11]. Recent studies in our laboratory have shown that the epicuticular wax crystals on Chinese kale leaves prevent DBM females from laying eggs on the plants. However, DBM larvae assist adult females by spinning silk to overcome the physical barrier of plant wax, enabling the females to successfully lay eggs on waxy plants [12]. Further research has revealed that DBM females can also utilize silk spun by heterospecific lepidopterous larvae to bypass the epicuticular wax barrier and complete egg-laying [13]. These findings significantly enhance our understanding of the role of insect silk and may represent an adaptive mechanism that DBM has evolved to cope with waxy host plants.

Silk production in insects is a complex process regulated by multiple genes [14]. Research on insect silk has primarily focused on *Bombyx mori*, with efforts directed toward enhancing silk yield and improving its mechanical properties to meet growing application demands [15,16]. In contrast, the mechanisms and characteristics of silk production in other silk-producing insects have not been extensively studied. Studies have indicated that the 20-hydroxyecdysone gene suppresses the expression of silk protein genes in DBM, thus shortening its cocoon-spinning duration [17]. The silk produced by *B. mori* consists mainly of fibroins and sericins: sericins are synthesized in the middle silk gland and provide a sticky covering over the fibroins [18,19,20]; fibroins, produced in the posterior silk gland, consist of a heavy chain (FibH), a light chain (FibL), and fibrohexamerin (P25), all of which are essential for fibroin formation [21,22,23]. Recent advancements in understanding the regulatory mechanisms governing these proteins have been achieved through transcriptomic and proteomic analyses, which have revealed the temporal regulation of silk fibroin protein synthesis and the structural characteristics of the silk gland in *B. mori* [24,25]. Additionally, comparative genomic studies across various silk-producing insects have begun to uncover the evolutionary dynamics and differentiation of silk protein genes [26,27]. These findings significantly enhance our understanding of silk production in different insect species.

In our transcriptomic analysis of DBM, we identified three analogous genes: *PxFibH*, *PxFibL*, and *PxP25*. However, their genetic structures and physicochemical properties remain poorly characterized. To address this gap, we identified and characterized these silk fibroin genes through comprehensive transcriptomic analysis, examining their structural features and physicochemical parameters, including isoelectric point (pI), molecular weight (Mw), hydrophilicity, and amino acid composition. Furthermore, we performed phylogenetic and spatio-temporal expression analyses. These findings significantly advance our understanding of silk fibroins in DBM and provide a solid foundation for future studies on the regulatory mechanisms underlying DBM silk production.

## 2. Results

### 2.1. Overview of RNA-Seq Data

Transcriptomic analyses yielded high-quality RNA sequencing data for all samples. A total of 397,201,388 raw reads were generated from three biological replicates of silk glands (SG), heads (H), and residual body parts (RB). After data processing, 97.52% (387,367,380/397,201,388) of the clean reads were retained, with base quality percentages exceeding 90% for both Q20 and Q30 (Table 1). These results indicate that the sequence data quality is adequate for subsequent gene expression analyses.

### 2.2. Transcriptome Sequencing Analysis

**Figure 1:**
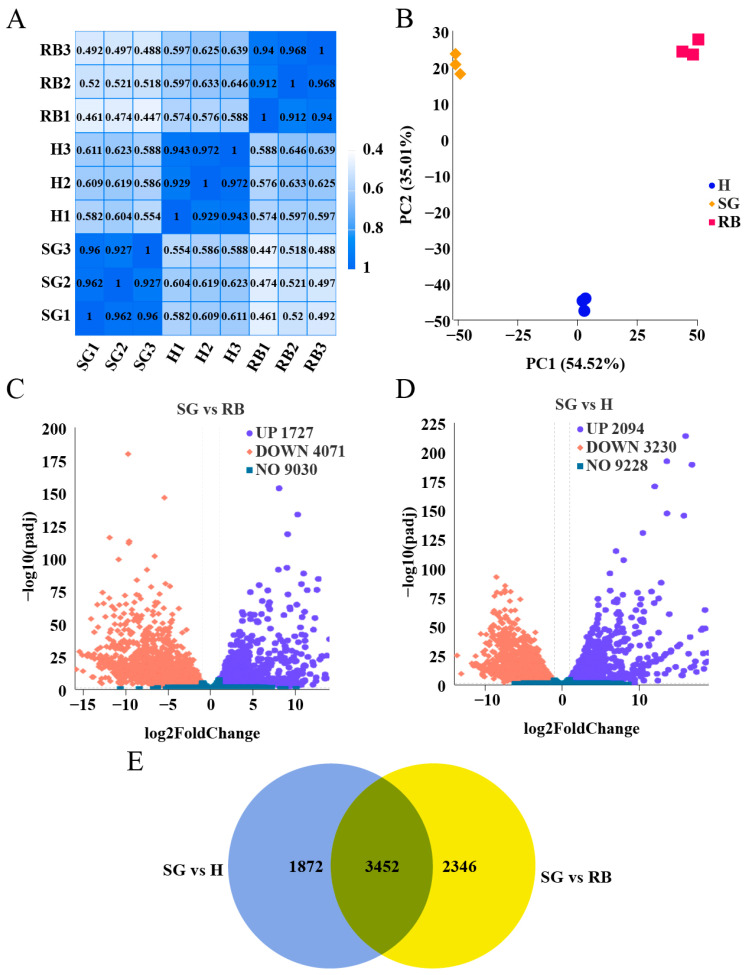
Transcriptome analysis in the silk gland. (**A**) Pearson correlation between different samples. (**B**) Principal component analysis between different samples. (**C**) Differential expressed genes in the silk gland, using the residual body as a control. (**D**) Differential expressed genes in the silk gland, using the head as a control. (**E**) Overlap of differentially expressed genes. SG: silk gland; H: head; RB: residual body.

Pearson correlation analysis (PCA) of the gene expression values across all samples demonstrated that the squared Pearson’s coefficients (R^2^) for biological replicates within each group exceeded 0.8 (Figure 1A). PCA further illustrated that samples were more similar within groups than between groups (Figure 1B). Quantitative gene expression analysis revealed 1727 up-regulated and 4071 down-regulated differentially expressed genes (DEGs) in the SGvsRB comparison (Figure 1C). In the SGvsH comparison, 2094 up-regulated and 3230 down-regulated DEGs were identified (Figure 1D). A total of 3452 DEGs were found to be co-expressed across these comparisons (Figure 1E).

### 2.3. GO and KEGG Enrichment Analysis

**Figure 2:**
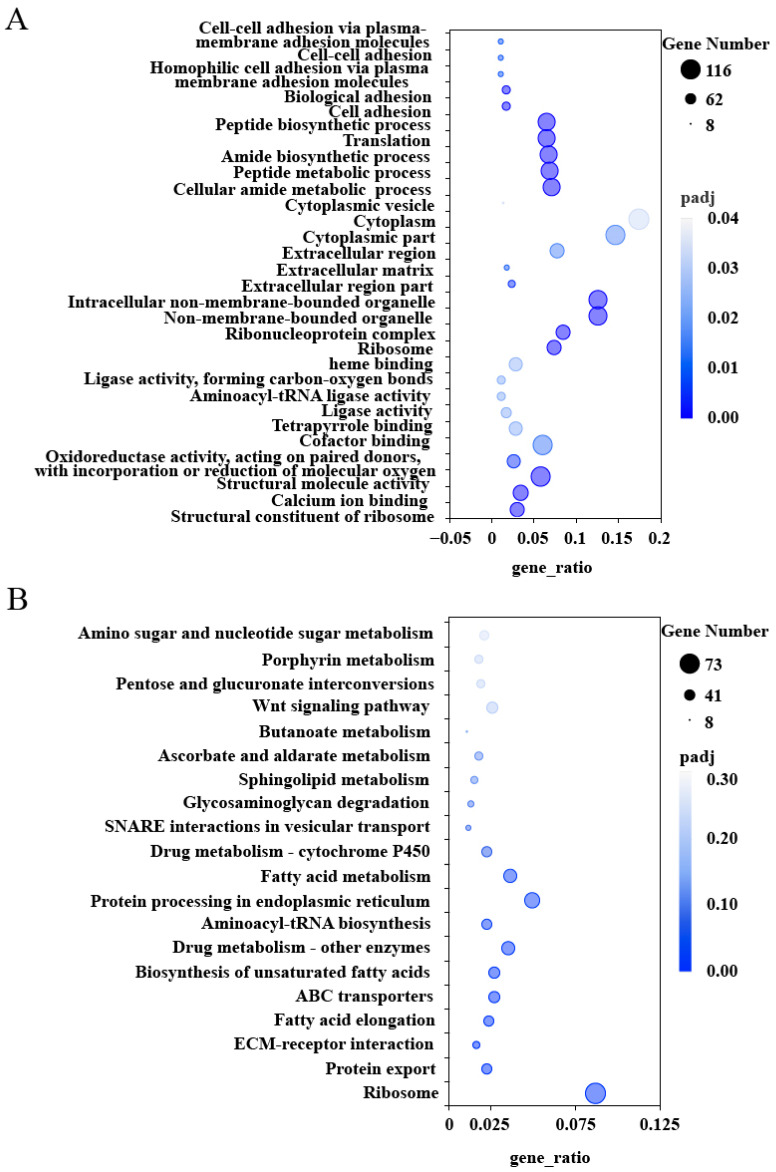
GO (**A**) and KEGG (**B**) enrichment analysis of differentially expressed genes. Gene_ratio represents the proportion of differentially expressed genes annotated to the KEGG pathway relative to the total number of significantly differentially expressed genes.

Gene Ontology (GO) and Kyoto Encyclopedia of Genes and Genomes (KEGG) enrichment analyses were conducted on the 3452 co-expression DEGs, with significance determined by Padj ≤ 0.05. In the silk gland, the most significantly enriched GO terms included Cellular amide metabolic process, Peptide metabolic process, Amide biosynthetic process, Translation, Non-membrane-bounded organelle, Peptide biosynthetic process, Calcium ion binding, Cofactor binding, Cytoplasm, and Structural molecule activity (Figure 2A). At the KEGG pathway level, the DEGs were predominantly enriched in the following pathways: Ribosome, Protein export, ECM–receptor interaction, Fatty acid elongation, ABC transporters, Biosynthesis of unsaturated fatty acids, Protein processing in the endoplasmic reticulum, and Fatty acid metabolism (Figure 2B).

### 2.4. RT-qPCR Validation

**Figure 3:**
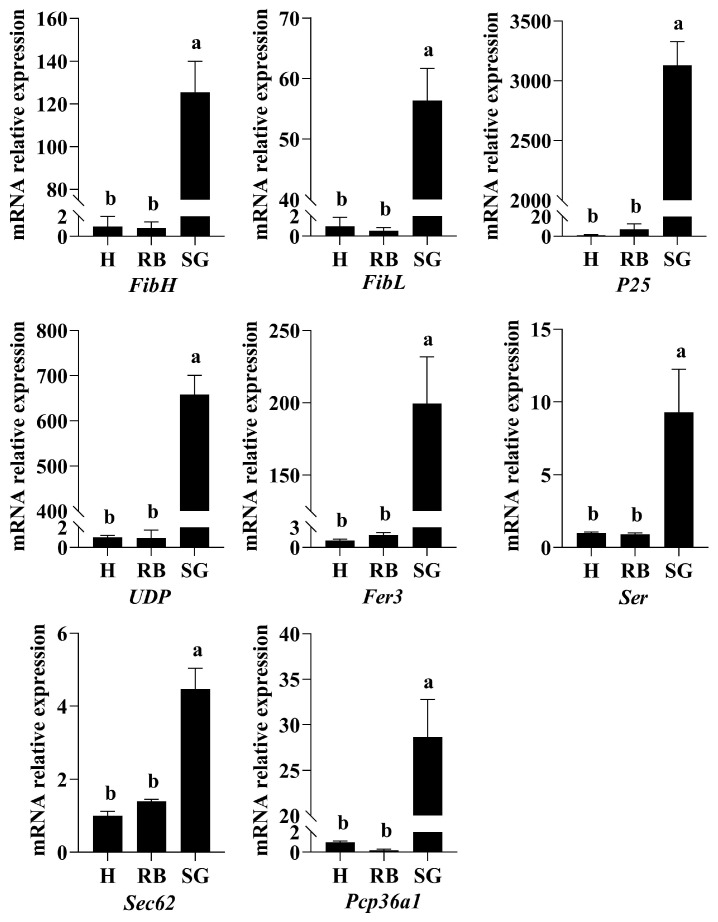
RT-qPCR validation of RNA-Seq. Data are presented as mean ± SEM. Different lowercase letters indicate significant differences at *p* < 0.05. SG: silk gland; H: head; RB: residual body.

To validate the RNA-seq findings, eight up-regulated DEGs were randomly selected for RT-qPCR analysis. The genes examined included *FibH* (fibroin heavy chain), *FibL* (fibroin light chain), *P25* (fibrohexamerin), *UDP* (UDP-glycosyltransferase), *Fer3* (fer3-like protein), *Ser* (serine-tRNA ligase), *Sec62* (translocation protein SEC62), and *Pcp36a1* (pupal cuticle protein 36a1). The relative expression levels obtained from RT-qPCR corroborated the trends observed in the RNA-seq analysis, thereby confirming the reliability of the transcriptomic data (Figure 3).

### 2.5. Silk Fibroins Analysis and Phylogenetic Tree Construction

**Figure 4:**
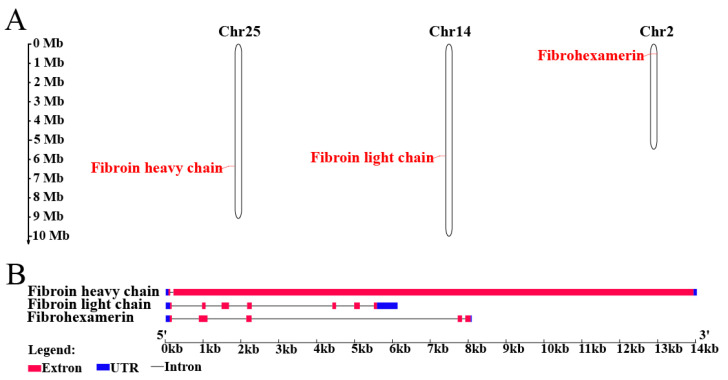
Chromosomal location and genomic structure of silk fibroin genes. (**A**) Distribution of silk fibroin genes across the chromosomes. (**B**) Genomic structure of the silk fibroin genes.

**Figure 5:**
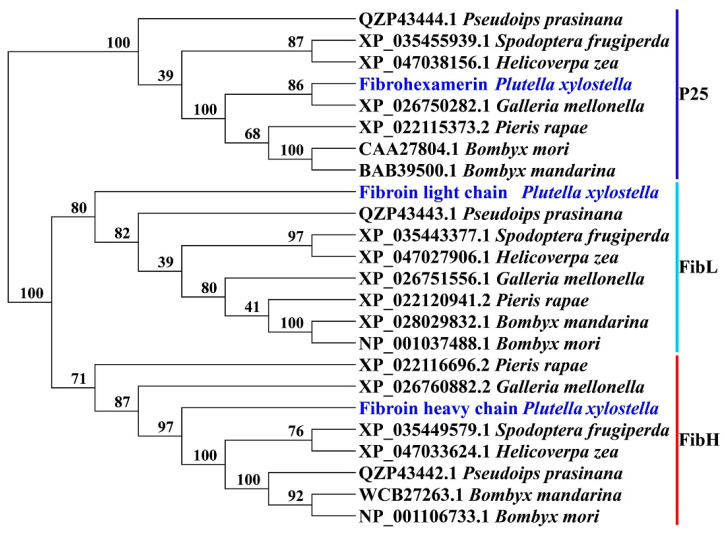
The phylogenetic tree of silk fibroin proteins in *P. xylostella*. Bootstrap values from 1000 replicates are displayed at each node.

The genes *PxFibH*, *PxFibL*, and *PxP25* are located on chromosomes 25 (14.0 kb), 14 (6.1 kb), and 2 (8.1 kb), respectively (Figure 4A). *PxFibH* consists of two exons, while *PxFibL* comprises seven exons, and *PxP25* contains five exons (Figure 4B). A detailed comparison of their sizes, isoelectric points, and chromosomal locations with those of other Lepidoptera species is provided (Table S1). Phylogenetic analysis indicates that the silk fibroin proteins of *P. xylostella* exhibit a close relationship with those of other Lepidopteran species. Notably, FibH and FibL proteins form sister clades that further cluster with the P25 protein, resulting in a monophyletic group. Interestingly, the P25 protein of *P. xylostella* clusters with that of *G. mellonella*, whereas the FibH and FibL proteins of *P. xylostella* form a distinct clade (Figure 5).

### 2.6. Amino Acid Composition and Hydrophilicity

**Figure 6:**
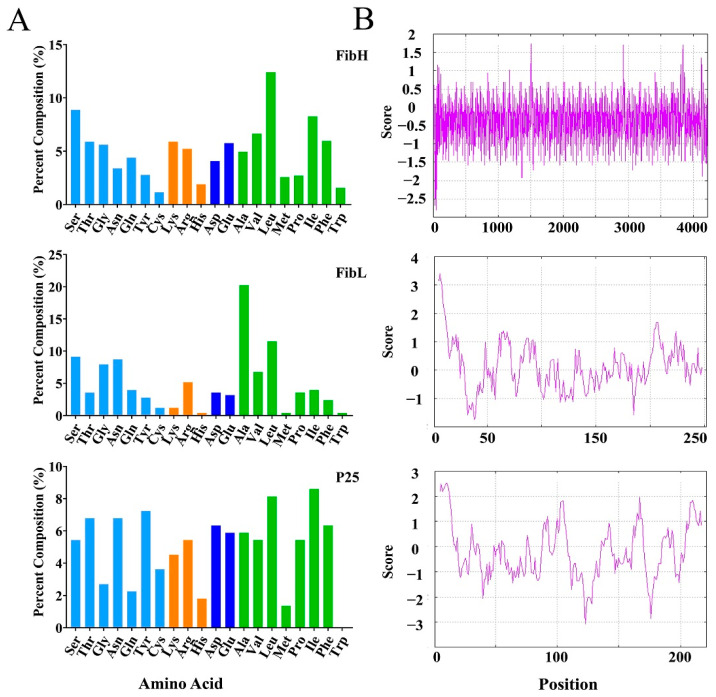
Comparison of amino acid composition and hydrophobicity of fibroin proteins. (**A**) Percent composition analysis of amino acids in fibroin proteins. Neutral amino acids are highlighted in light blue, basic amino acids in orange, acidic amino acids in dark blue, and nonpolar amino acids in green. (**B**) Kyte and Doolittle hydrophobicity plots for fibroin proteins.

Analysis of the predicted amino acid properties categorizes the three proteins into four types: neutral, basic, acidic, and non-polar amino acids. The amino acid compositions of FibH and FibL display similar patterns, with leucine as the most abundant amino acid in FibH and alanine as the most prevalent in FibL. Both FibH and P25 exhibit a higher percentage of polar amino acids compared to FibL. In contrast, P25 lacks tryptophan, and its amino acid composition shows relatively consistent proportions across the various categories (Figure 6A). Furthermore, hydrophilicity predictions revealed that FibH exhibits the highest hydrophilicity, followed by P25, while FibL demonstrates the greatest hydrophobicity (Figure 6B).

### 2.7. Expression Profile of Silk Fibroin Genes

**Figure 7:**
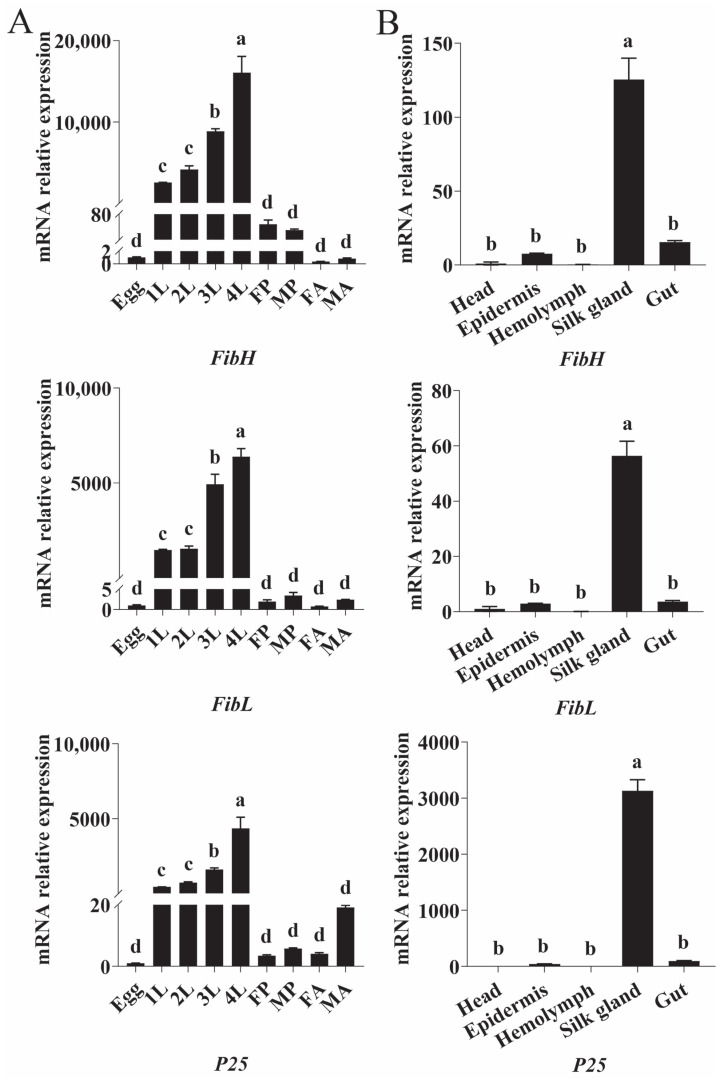
Expression profiles of silk fibroin proteins at different developmental stages (**A**) and in various tissues (**B**) of *P. xylostella*. 1–4 L: first to fourth instar larvae; F/MP: female/male pupae; F/MA: female/male adults. Data are presented as mean ± SEM. Different lowercase letters indicate significant differences at *p* < 0.05.

The developmental expression profiles of *PxFibH* (*F*_8,18_ = 64.91, df = 26, *p* < 0.001), *PxFibL* (*F*_8,18_ = 109.60, df = 26, *p* < 0.001), and *PxP25* (*F*_8,18_ = 34.77, df = 26, *p* < 0.001) exhibit a consistent pattern: the expression levels of all three genes increase with the age of DBM, peaking during the fourth larval instar, followed by a rapid decline (Figure 7A). To further investigate, we assessed the expression profiles of these genes across different tissues. The results demonstrated that all three genes are highly specifically expressed in the silk gland of DBM (*FibH*: *F*_4,10_ = 67.82, df = 14, *p* < 0.001; *FibL*: *F*_4,10_ = 103.70, df = 14, *p* < 0.001; *P25*: *F*_4,10_ = 245.50, df = 14, *p* < 0.001) (Figure 7B).

## 3. Discussion

Cocoon formation plays a crucial role in the survival and reproduction of many insect species. In particular, the cocoons of various Lepidoptera species contain silk fibroin, which is composed of FibH, FibL, and P25 proteins [22,28,29,30]. This study provides a comprehensive analysis of DEGs in the silk glands of DBM using transcriptomic techniques to identify key genes involved in silk production. Among the identified DEGs, three silk fibroin genes–*PxFibH*, *PxFibL*, and *PxP25*–emerged as pivotal players in the silk formation process. Our findings confirm previous studies on silk fibroin genes in other Lepidoptera species and provide new insights into the molecular mechanisms underlying silk production in DBM.

RNA-seq analysis identified a total of 3452 co-expressed DEGs in the silk glands of DBM (Figure 1), with significant enrichment in pathways related to ribosome function, calcium ion binding, protein export, and the biosynthesis of unsaturated fatty acids (Figure 2). These pathways are crucial for the synthesis and secretion of silk proteins, as they facilitate the production and transport of silk proteins [31]. Notably, *PxFibL* and *PxP25* were enriched in GO terms related to the extracellular region and structural molecule activity (Figure 2A). These enrichments align with the known functions of these genes, which encode proteins that form the structural backbone of silk fibroin and are secreted into the extracellular space during cocoon formation [21]. Although *PxFibH* plays a pivotal role in silk production and is highly expressed in the silk glands, it was not enriched in any GO terms. This may be due to the highly specialized function of *PxFibH*, which focuses primarily on the synthesis and translation of silk proteins, rather than being broadly involved in multiple biological processes [32]. In contrast, none of the three silk fibroin genes were enriched in KEGG pathways (Figure 2B). This finding is somewhat surprising, given the critical role of these genes in silk production. The KEGG database mainly focuses on metabolic pathways, signal transduction, and disease-related genes [33], while the silk fibroin genes, which are responsible for encoding silk proteins, are more closely involved in the synthesis of biological materials rather than typical metabolic or signal transduction pathways [22]. As a result, these genes may not be enriched in KEGG, which is dominated by metabolic pathway data. Despite these limitations, our findings provide valuable insights into the complex transcriptional regulation of silk gland function and emphasize the critical role of silk fibroin genes in silk production.

In this study, we explored the silk fibroin genes in DBM, revealing their chromosomal localization across chromosomes 25, 14, and 2, respectively (Figure 4A). This finding aligns with the independent evolution of these genes within the DBM genome. The distribution of these three silk fibroin genes is identical to that observed in *B. mori* [34]. The variation in exon number among these genes suggests distinct regulatory mechanisms and structural adaptations that influence protein synthesis (Figure 4B), potentially contributing to differences in the mechanical and biochemical properties of silk fibroins. While the structure of silk fibroin genes is generally conserved across most Lepidoptera, significant variation was observed in Saturniidae silkmoths, including modified FibH and the absence of FibL and P25 [35,36]. Phylogenetic analysis demonstrates that the silk fibroins of DBM share a close evolutionary relationship with those of other Lepidopteran species, underscoring the conserved nature of silk production across this order (Figure 5). Notably, FibH and FibL form sister clades, suggesting that they diverged from a common ancestral gene and underwent functional differentiation. In contrast, P25 appears to maintain a more conserved role in the silk biosynthetic pathway. These findings provide valuable insights into the molecular evolution of silk production in DBM, highlighting both the evolutionary conservation and diversification of silk fibroins in Lepidoptera.

FibH and FibL proteins, the primary components of silk fibroins, exhibit distinct amino acid composition patterns that are crucial for the formation of strong yet elastic silk fibers [37,38]. Our analysis reveals that FibH is predominantly composed of leucine, while FibL is rich in alanine (Figure 6A). These differences likely contribute to the unique physicochemical properties of the silk fibroins [39]. In contrast, P25, which is also involved in silk formation, lacks tryptophan, suggesting that it may serve a distinct biological function compared to FibH and FibL. Tryptophan plays a vital role in various biological processes, including enzymatic activity [40], signal transduction [41], and metabolism [42], so its absence in P25 may indicate a unique biological mechanism [43]. Additionally, hydrophilicity analysis (Figure 6B) revealed that FibH is the most hydrophilic, while FibL is the most hydrophobic. This contrast highlights the importance of amino acid composition in determining the solubility and structural integrity of silk proteins [44]. These differences in amino acid composition reflect the specialized roles of each silk fibroin protein in silk formation, indicating their unique biological functions and mechanisms.

The expression profiles of the silk fibroin genes, peaking during the fourth larval instar (Figure 7A), align with the timing of cocoon formation, suggesting that these genes are tightly regulated to support the physiological demands of silk production during this critical stage. The high expression of these genes in the silk gland (Figure 7B) further emphasizes their crucial role in silk production. Notably, longitudinal analysis revealed a progressive upregulation of *PxFibH*, *PxFibL*, and *PxP25* expression across successive larval instars, forming a coordinated expression cascade that culminates in maximal silk protein synthesis prior to pupation. This developmental trajectory coincides with the increasing silk output required for constructing complete cocoons in later instars, a pattern consistent with observations in *Corcyra cephalonica* and *B. mori* [45,46,47,48]. While previous studies in DBM primarily focused on the fourth instar expression peaks [17], the expanded developmental profiling identified two distinct regulatory phases. The first phase, characterized by basal expression during early larval stages, likely supports the structural maintenance of the silk glands. The second phase, marked by exponential upregulation starting from the third instar, meets the biophysical demands of cocoon spinning. This biphasic regulation provides new insights into how silk production capacity is developmentally programmed in Lepidoptera.

DBM, a destructive pest of cruciferous vegetables worldwide, is recognized as one of the most resistant agricultural pests [49]. Its strong environmental adaptability, short generation time, and high reproductive capacity make its prevention and control particularly challenging [50]. Our findings suggest that the three key silk fibroin genes, *PxFibH*, *PxFibL*, and *PxP25*, play essential roles in the DBM life cycle. By utilizing gene editing technologies such as CRISPR/Cas9, we could potentially reduce silk production in DBM, compromising its defensive mechanisms and survival. This reduction may increase the vulnerability of larvae to predators and environmental stressors, ultimately decreasing population density and migration. Additionally, RNA interference (RNAi) technology could be employed to develop RNAi-based biopesticides for pest management [51]. These approaches hold potential for the development of innovative pest control technologies for DBM.

## 4. Materials and Methods

### 4.1. Insect Culture

A DBM culture was maintained in a greenhouse under controlled conditions of 25 ± 1 °C, 60 ± 5% relative humidity, and a 16 L:8 D photoperiod since its collection in 2017 from Yangling, Shaanxi, China. The larvae were reared on Chinese kale (*Brassica oleracea* var. alboglabra Bailey cv. Zhonghuajianye). For RNA-sequencing experiments, three biological replicates were prepared, each consisting of 100 pairs of silk glands. The heads and residual body parts of the moths were used as control samples. Silk glands were carefully dissected by tearing the tissue at the junction between the larval thorax and head. The head and end of the abdomen were grasped with forceps and gently pulled to extract the complete silk glands. Specimens for RT-qPCR were collected from pooled insects across various developmental stages, including eggs, first to fourth instar larvae, male and female pupae, and adult males and females. During the fourth larval instar, five tissues were harvested: the head, hemolymph, silk glands, intestine, and integument. All samples were rapidly frozen in liquid nitrogen and stored at −80 °C for subsequent analysis. Each sample was replicated three times to ensure reproducibility.

### 4.2. RNA Sequencing and Analysis

Total RNA was extracted using TRIzol™ reagent (Invitrogen, Waltham, MA, USA), according to the manufacturer’s instructions. RNA integrity was assessed using the RNA Nano 6000 Assay Kit (Agilent Technologies, Santa Clara, CA, USA) on the Bioanalyzer 2100 system (Agilent Technologies, Santa Clara, CA, USA). RNA sequencing (RNA-seq) libraries were prepared with the NEBNext^®^ Ultra^TM^ RNA Library Prep Kit (New England Biolabs, Ipswich, MA, USA), following the manufacturer’s protocol. RNA-seq was conducted at Novogene Technology (Beijing, China) on the Illumina NovaSeq 6000 (Illumina, San Diego, CA, USA) sequencing platform, generating 150 bp paired-end reads. Following sequencing, fastp software (version 0.19.7, parameters: fastp -g -q 5 -u 50 -n 15 -I 150) was used to remove reads containing adapters, ploy-N sequences, and low-quality data. Reads with a quality score below 5 were filtered out, and sequences shorter than 50 bp after trimming were discarded. Additionally, duplicate reads were also removed using the deduplication function in fastp, which identifies and retains only unique sequences. Clean reads were then aligned to the reference genome of DBM (GCF_932276165.1) using Hisat2 (version 2.0.5) with default parameters to obtain genetic information for further analysis. Raw data were uploaded in fastq format and stored in the Sequence Read Archive (SRA) in NCBI under accession PRJNA1141711.

Gene expression levels were calculated as fragments per kilobase of transcript per million mapped reads (FPKMs). Sample correlation heatmaps and PCA were used to evaluate inter-group differences and intra-group reproducibility. Differentially expressed genes were identified using DESeq2 (version 1.20.0), which internally normalized the raw read counts. Significance thresholds were set at Padj < 0.05 and |log2(fold change)| ≥ 1.

### 4.3. Bioinformatic Analysis

Three candidate transcript sequences were submitted to NCBI (Accession number: PQ136835, PQ136836, PQ136837). Chromosomal positions were predicted using the MapGene2chrom tool (http://mg2c.iask.in/mg2c_v2.1, accessed on 8 May 2024) based on the *Plutella xylostella* reference genome (GCF_932276165.1) [52]. Signal peptides were identified using the SignalP 5.0 Server (https://services.healthtech.dtu.dk/services/SignalP-5.0, accessed on 9 November 2022) [53]. The theoretical pI and Mw were calculated using the Compute pI/Mw tool (web.expasy.org/compute_pi, accessed on 5 December 2023) [54]. Average hydropathy values were determined using the Gravy Calculator (https://www.detaibio.com/sms2/protein_gravy.html, accessed on 5 December 2023). The Kyte & Doolittle hydropathy profile was plotted with the Protscale tool (web.expasy.org/protscale, accessed on 15 June 2024) [55]. Amino acid composition was analyzed using the Protein Stats tool (https://www.detaibio.com/sms2/protein_stats.html, accessed on 6 October 2024) [56].

### 4.4. Phylogenetic Analysis

To perform a comparative analysis of FibH, FibL, and P25 protein sequences across various species, sequences from *Galleria mellonella*, *Spodoptera frugiperda*, *Helicoverpa zea*, *Bombyx mori*, *Bombyx mandarina*, *Pieris rapae*, and *Pseudoips prasinana* were downloaded from the NCBI database. These sequences were aligned using MEGA 11 software for multiple sequence alignment. A phylogenetic tree was constructed using the Neighbor-Joining method with a bootstrap value of 1000, following standard protocols [57]. The Jukes–Cantor model was used as the substitution model, and the distance matrix was calculated based on pairwise deletion of gaps/missing data. The ChiPlot online tool was used to enhance the clarity and visual presentation of the tree [58].

### 4.5. RNA Preparation and RT-qPCR

Total RNA was extracted from the samples using TRIzol™ reagent (Invitrogen, Waltham, MA, USA), according to the manufacturer’s instructions. RNA quality was assessed using a NanoDrop2000 spectrophotometer (Thermo Fisher Scientific, Wilmington, DE, USA), and RNA integrity was further verified by 1.5% agarose gel electrophoresis. Reverse transcription was performed using the PrimeScript™ RT Kit with gDNA Eraser (Takara, Kusatsu, Japan). For gDNA removal, 2 µL of 5× gDNA Eraser Buffer, 1 µL of gDNA Eraser, 1000 ng of total RNA template, and RNase-free water were mixed to a final volume of 10 μL and incubated at 42 °C for 2 min. For reverse transcription, 1 µL of PrimeScript RT Enzyme Mix I, 1 µL of RT Primer Mix, 4 µL of 5× PrimeScript Buffer 2, and 4 µL of RNase-free water were added, and the reaction was incubated at 37 °C for 15 min, followed by 85 °C for 5 s. A final volume of 20 µL of cDNA was obtained for subsequent analysis. The RT-qPCR reaction mix included 5 μL of TB Green Premix Ex Taq (Tli RNaseH Plus) (2×, TaKaRa, Kusatsu, Japan), 0.2 μL of each primer (10 μM), 1 μL of 10× diluted cDNA, and RNase-free water to a final volume of 10 μL. Real-time qPCR reactions were performed on a LightCycler 480 Real-Time PCR System (Roche, Basel, Switzerland) using a two-step cycling protocol: initial denaturation at 95 °C for 30 s, followed by 40 cycles of 95 °C for 5 s and 60 °C for 20 s. Each RT-qPCR assay included three biological replicates and three technical replicates. The relative expression levels of the target genes were calculated using the 2^−ΔΔCt^ method, normalized to the ribosomal protein L32 (*RPL32*) gene [17]. Differences in relative gene expression were analyzed by comparing ΔCt values using one-way ANOVA. All primers used in this study are listed in Table 2.

## 5. Conclusions

In conclusion, this study elucidates the roles of silk-related genes in protein synthesis, secretion, and amino acid metabolism through GO and KEGG enrichment analysis. We identified and characterized three key silk fibroin genes (*PxFibH*, *PxFibL*, and *PxP25*) using transcriptomic data, providing detailed information on their structures, phylogenetic relationships, amino acid compositions, and physicochemical properties. This research advances our understanding of the molecular mechanisms underlying silk production in *P. xylostella*, establishing a strong foundation for future investigations into the biological functions, evolutionary trajectories, and potential applications of these genes in agricultural pest management and biotechnology. Notably, these silk fibroin genes represent viable targets for pest control strategies, where gene editing technologies could be employed to reduce silk production in pests, thereby weakening their defensive capabilities and overall fitness.

## Figures and Tables

**Table 1:** Summary of trimming and read mapping results for RNA-seq.

Sample	Raw Reads	Raw Bases	Clean Reads	Clean Bases	Q20	Q30	GC (%)
SG1	41,191,356	6.18 G	39,767,162	5.97 G	97.41	93.26	51.49
SG2	48,942,932	7.34 G	47,060,438	7.06 G	96.99	92.46	51.10
SG3	41,824,838	6.27 G	40,723,700	6.11 G	96.71	91.89	50.45
H1	42,368,122	6.36 G	41,705,096	6.26 G	97.47	93.28	48.56
H2	46,174,644	6.93 G	45,491,500	6.82 G	97.32	92.87	47.77
H3	42,519,786	6.38 G	41,719,820	6.26 G	97.53	93.38	48.42
RB1	46,530,060	6.98 G	45,525,936	6.83 G	97.28	92.97	52.93
RB2	44,564,120	6.68 G	43,548,404	6.53 G	97.54	93.48	51.42
RB3	43,085,530	6.46 G	41,825,324	6.27 G	97.48	93.48	51.76

SG: silk gland; H: head; RB: residual body.

**Table 2:** Primers for RT-qPCR.

Primers	Direction	Primer Sequence (5′-3′)
FibH	Forward	GCTCAAGTTCCGCAGCAACCA
	Reverse	CCCGCAGCAGCAGTGTTTGAA
FibL	Forward	CACACTAACCGCCGTGAACGT
	Reverse	GCCAGCAGGTTGATGGTCTGTC
P25	Forward	CCCGCCGAAATCTGCAATCCA
	Reverse	CGTTGGCGTTGCATCTGGAGTT
Udp	Forward	TGGCAACAGTGGACCGAGATCA
	Reverse	GCAGCAACTTGACACAGTGGCA
Fer3	Forward	TGAGGTGGCGGATCACAGCAA
	Reverse	CCTCCACTCCAGAGCAGCATCA
Ser	Forward	GCTGGTGTCGTGCAGCAACT
	Reverse	CGTCGCCGCGTTCATCTTCTT
Sec62	Forward	TCGGCTTTGTGGCTTCTTTCTGG
	Reverse	CTCCTCCTCGTCGTCGGAATGT
Pcp36a1	Forward	TCGTCATTCTCGTCGTCCGTCA
	Reverse	GGTTCCTCGTCTCCGTTCTGGT
RPL32	Forward	CAATCAGGCCAATTTACCGC
	Reverse	CTGCGTTTACGCCAGTTACG

## Data Availability

Raw sequencing data were deposited in the NCBI Short Read Archive (SRA) BioProject PRJNA1141711.

## Supplementary Materials

The supporting information can be downloaded at https://www.mdpi.com/article/10.3390/ijms26072842/s1.
