# Supplementary material for: Comprehensive Transcriptomic Analyses of Silk-Associated Genes and Functional Characterization of Key Silk Fibroins in Plutella xylostella
Source: Int J Mol Sci. 2025 Mar 21;26(7):2842. doi: 10.3390/ijms26072842 (PMC11988815; doi:10.3390/ijms26072842)
Supplement: Supplementary file 1 [file ijms-26-02842-s001.zip › Supplementary Table S1.pdf]

Table S1. Size comparisons and bioinformatic analysis of the silk fibroins

silk fibroins	*Plutella xylostella*	*Spodoptera frugiperda*	*Helicoverpa zea*	*Spodoptera litura*	*Bombyx mori*	*Pieris rapae*
**Silk fibroin heavy chain (FibH)**						
gene length(kb)	14.0	11.0	14.8	1.7	17.8	15.0
number of exons	2	6	23	2	3	2
predicted molecular weight (kDa)	417.85	246.66	164.24	41.85	391.62	433.24
isoelectric point (pI)	3.74	4.59	7.02	9.34	4.13	4.14
chromosome localization	25	8	11	16	25	1
residues of signal peptide	18	18	18	17	21	18
**Silk fibroin light chain (FibL)**						
gene length(kb)	6.1	8.5	5.5	6.5	13.3	6.7
number of exons	7	7	7	7	7	7
predicted molecular weight (kDa)	25.74	26.88	26.72	26.63	27.76	27.90
isoelectric point (pI)	5.91	4.93	8.02	4.91	5.19	8.01
chromosome localization	14	9	14	18	14	6
residues of signal peptide	16	16	16	16	16	16
**Fibrohexamerin (P25)**						
gene length(kb)	8.1	6.4	3.8	3.3	3.0	5.9
number of exons	5	5	5	5	5	5
predicted molecular weight (kDa)	25.62	29.47	25.62	26.83	25.17	26.42
isoelectric point (pI)	5.41	8.48	8.90	6.70	6.69	5.68
chromosome localization	2	22	2	10	2	16
residues of signal peptide	16	19	18	19	17	NO
